# Primary ovarian serous carcinomas with extensive squamous differentiation: a case report and literature review

**DOI:** 10.1186/s12905-021-01336-y

**Published:** 2021-05-08

**Authors:** Yao Sun, Yuezhou Chen, Xiaofei Zhang, Hao Chen, Feng Zhou

**Affiliations:** 1 Department of Pathology, School of Medicine, Women’s Hospital, Zhejiang University, Hangzhou, Zhejiang Province China; 2 Department of Reproductive and Genetic Medicine Center, The Fifth Affiliated Hospital of Sun Yat-Sen University, Zhuhai, China; 3 Department of Pathology, University of Texas Southwestern Medical Center, Dallas, Texas USA; 4 Departments of Pathology, School of Medicine, Women’s Hospital, Zhejiang University, Hangzhou, Zhejiang Province China

**Keywords:** Ovary, Serous carcinoma, Squamous differentiation

## Abstract

**Background:**

Primary ovarian serous carcinomas (OSC) with extensive squamous differentiation is a rare, and histological diagnostic criteria and biological behavior have not been fully established. We present an extremely rare case of primary OSC of the ovary with squamous differentiation.

**Case presentation:**

A 58-year-old (gravidity 3, parity 2) female was admitted complaining of abdominal distention for 6 months. No apparent tumor in the cervix was found by a physical examination. Serum levels of cancer antigen 125 (CA125) was elevated (2723.0 IU/L). Macroscopically, a 7 cm tumor of the left uterine adnexa, a 5 cm tumor of the right adnexa, and a 3 cm tumor of the omentum were found. Histological and immunochemical tests confirmed a diagnosis of OSC with squamous differentiation. Debulking surgery with tumor resection was performed. The patient was subsequently received postoperative chemotherapy.

**Conclusions:**

In summary, OSC with extensive squamous differentiation is a rare, and the inter- and intratumor heterogeneity may be the reason for this phenomenon. Histological diagnostic criteria and biological behavior have not been fully established because of the limited data.

## Background

Squamous differentiation is frequently present in endometrioid adenocarcinoma, but not in certain varieties of ovarian epithelial tumors, notably serous types. As far as we know, only three published cases of serous carcinoma with squamous differentiation were found [1]. In the present study, we report a rare case of ovarian serous carcinoma (OSC) with extensive squamous differentiation, which was confirmed by immunohistochemical staining.

## Case presentation

A 58-year-old (gravidity 3, parity 2) female was admitted complaining of abdominal distention for 6 months. No apparent tumor in the cervix was found by a physical examination. Thinprep cytologic test and HPV E6/E7 mRNA (tested by Aptima assay) were negative in the cervix. Her serum level of cancer antigen 125 (CA125) was elevated (2723.0 IU/L). Her serum carcinoembryonic antigen and CA19-9 levels were in normal. Computed tomography identified a tumor on the left uterine adnexa, a tumor in the right adnexa and a tumor from the omentum. Pelvic and periaortic lymph nodes were normal. Thus, surgical treatment was suggested.

Macroscopically, the uterus measuring 9.0 × 6.0 × 5.0 cm with a normal endometrium. A 7 cm tumor on the left uterine adnexa, a 4 cm tumor of the right adnexa, and a 5 cm tumor of the omentum were evident. Other tumors (0.5-3 cm) in the mesentery, omentum, and paracolic sulcus were also detected. On cutting, the ovarian tumor surface was predominantly solid and gray in color, accompanied by obvious hemorrhage and necrosis. No cervical mass was apparent.

**Fig. 1:**
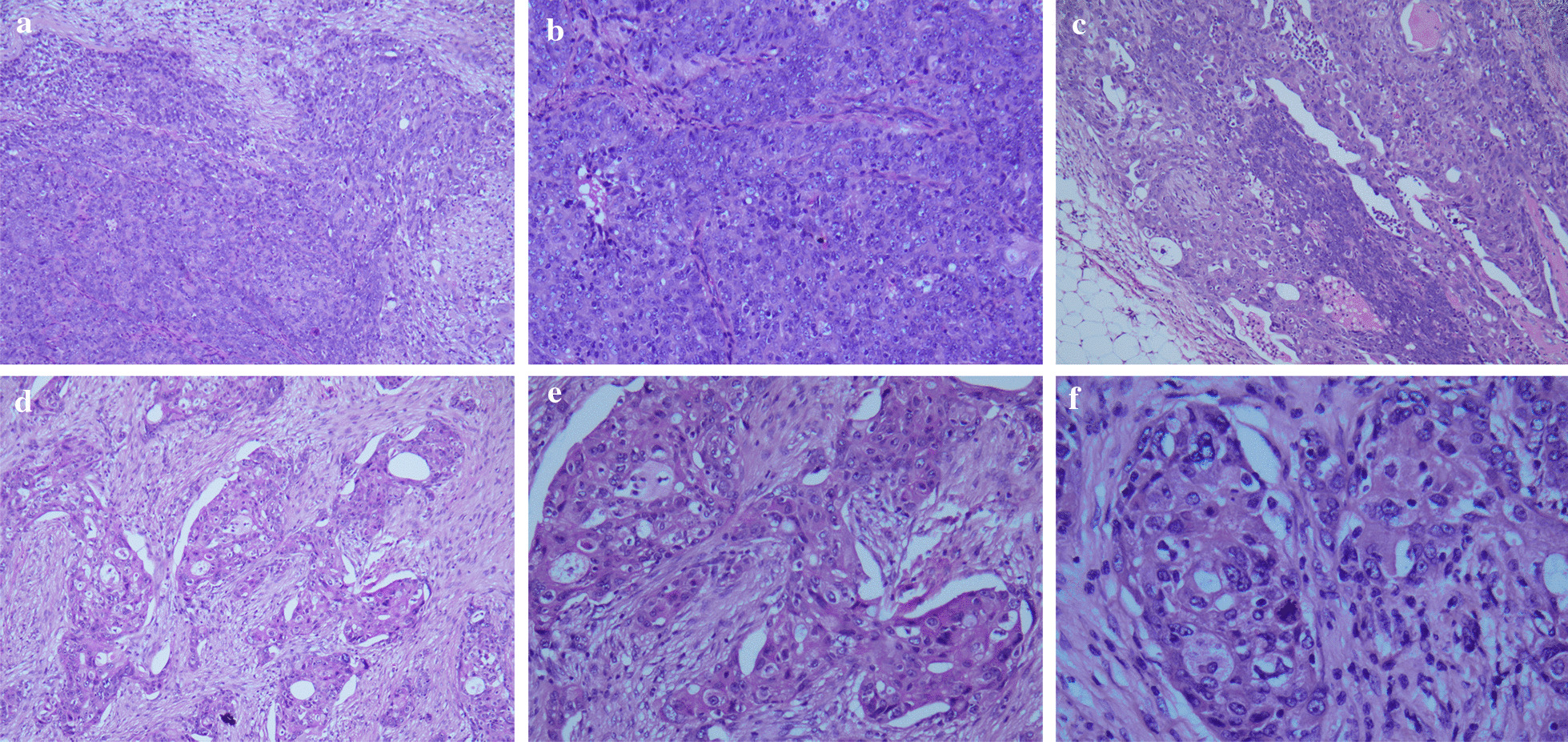
The tumors showed an infiltrative pattern of stromal invasion growing in sheets or nests. Representative area of serous differentiation: these atypical cells had large round to oval nuclei (**a**, 10×, **b**, 20×). Representative area of ovarian serous carcinomas with squamous differentiation: the tumor cells had severe nuclear pleomorphism with eosinophilic cytoplasm and numerous mitotic figures (**c**, 10×, **d**, 10×, **e **×20, **f **×40)

Microscopically, the ovarian tumors showed poorly defined islands growing in sheets or nests with numerous mitotic figures. Some tumor cells had marked pleomorphism and bizarre forms with high nuclear to cytoplasmic ratio and prominent nucleoli; others had severe nuclear pleomorphism with eosinophilic cytoplasm. Nuclei were pleomorphic in size and shape, and often had one or two prominent central nucleoli (Fig. 1).

**Fig. 2:**
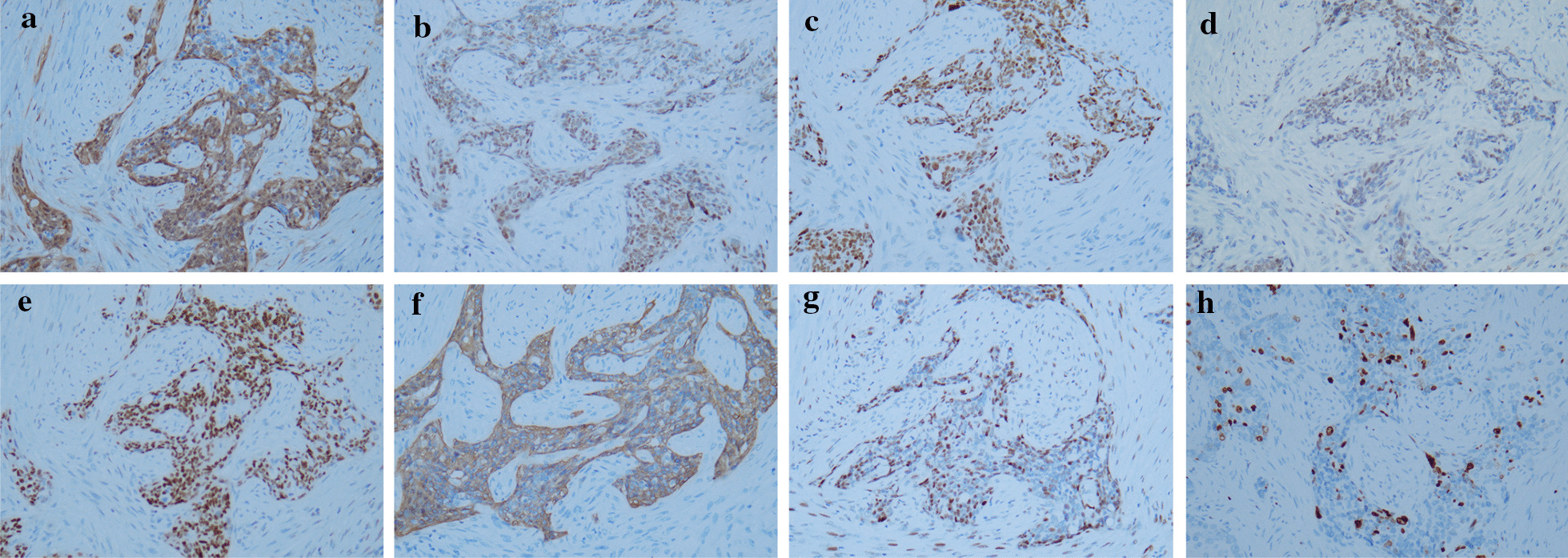
The immunochemical photograph of ovarian serous carcinomas with squamous differentiation. p16 (**a**, 20×), paired box 8 (**b**, 20×), p53 (**c**, 20×), Wilms tumor 1 (**d**, 20×), estrogen receptor (**e**, 20×), cytokeratin 5/6 (**f**, 20×), p63 (**g**, 20×), Ki67 (**h**, 20×), stained in the tumor cells

Immunohistochemically, paired box 8 (PAX8), p16, p53 (mutations), Wilms tumor 1 (WT1), estrogen receptor (ER), p63, and cytokeratin 5/6 (CK5/6) was strongly positive and the Ki67 index significantly decreased in the squamous differentiation area (Fig. 2).

Taking into account the above features, we made the diagnosis of high-grade OSC with squamous differentiation. The patient was subsequently received postoperative chemotherapy.

## Discussion and conclusions

The surface epithelium of the ovary, which is considered as the source of common epithelial tumors of the ovary, can differentiate into squamous cells. Primary squamous cell carcinoma (SCC) of the ovary can occur as an endometriosis, teratoma, or Brenner’s tumor [2, 3]. However, we found no evidence of these in present study.

A typical area of OSC was confirmed, as demonstrated by its solid growth morphology, marked pleomorphism, and prominent nucleoli with numerous mitotic figures. However, the ovarian tumor described here bore some resemblance to SCC in terms of its morphological character. Thus, we used several immunohistochemical markers, such as PAX8, p16, p53, WT1, ER, p63, and CK5/6, to make a distinction between the primary OSC and ovarian metastatic SCC.

The immunohistochemistry p53/p16 index was a good marker for high-grade OSC, which are defined as tumors with diffuse p53 expression or complete absence of p53 expression (null type) associated with diffuse p16 expression [4]. Notably, p16 overexpression is more frequently found in serous carcinomas [5]. p53, a tumor suppressor gene, is typically overexpressed in OSC and is regarded as a useful marker of such carcinomas [4, 6]. TP53 gene mutations are present in nearly 100% of high-grade OSCs [4]. WT1 has been proved to play an important role in the normal development of the kidneys and gonads and more usually found in OSC. It’s location in the female genital tract is usually used to distinguish OSC from other ovarian tumor types [4, 7]. ER reactivity was demonstrated in almost all the OSC, but most of SCCs were negative for ER [8, 9]. Moreover, expression of PAX8 confirmed that the tumor originates from female genital tract, such as the ovaries, uterus, and fallopian tubes [10]. p63 and CK5/6 are typically expressed in human squamous epithelium and SCC, but not OSC [11].

In addition, endometrioid carcinoma should be excluded, where confirmatory endometrioid features include squamous differentiation can be present in about half of cases [12]. Most OSCs can readily be distinguished from endometrioid carcinoma by the presence of specific morphologic features and immunostain profile. PAX8, p53 (mutations), WT1, and p16 were positive in most ovarian tumor cells described herein, which is a typical feature of OSC. Interestingly, the tumor cells with severe nuclear pleomorphism and eosinophilic cytoplasm also showed intense cytoplasmic CK5/6 expression and strong nuclear p63 staining. Such a finding of immunohistochemical staining is sufficient to constitute evidence of both patterns of OSC and SCC. Interestingly, the Ki67 index significantly decreased in the squamous differentiation area, which indicates a decreased activity.

We reviewed the current English literature and found only 3 similar cases [1, 2]. The clinical and pathological features were summarized in Table 1. Ulbright et al. [1] first described two cases of OSC with squamous differentiation that were confirmed by the immunohistochemical markers 35βH11 and 34βE12, and by electron microscopy. SCC arising in association with an OSC was recently reported in a 72-year-old woman [2]. Histological and immunochemical studies have confirmed distinct areas of OSC and SCC, and several small foci of the tumor showing transitional features between serous and squamous differentiation may also be seen. Interestingly, OSC with extensive squamous differentiation occupied the main part of the tumor in our case, arguing for a metaplastic origin of one component from a subset of the original neoplasm. Given the serous morphology and immunohistochemical profile of regions, coupled with no evidence of endometriosis, teratoma, or Brenner’s tumor, we confirmed that the present case provides evidence of a high-grade OSC differentiating into an aggressive SCC.

**Table 1:** The clinical and pathological features of primary ovarian serous carcinomas with extensive squamous differentiation

Authors [References]	Age (yr)	Symptom	Figo stage	Transitional area	Immunostain	Treatment	Fllow-up
Thomas et al. [1]	63	Postmenopausal bleeding	IIB	70%	35βH11+, 34βE12+	TAH-BSO + OT + RT + CTX	Died at 4 yr
	46	Abdominal swelling	III	5%	35βH11+, 34βE12+	BSO + OT + CTX	Died at 2 yr
Daniel et al. [2]	61	Lower abdominal pain	IIIC	Foci	p16+, PAX8+, p53+, WT1+, ER+, CK5/6+, p63+	TAH-BSO + RT	Na
Present case	58	Abdominal fullness	IIIC	Extensive	p16+, PAX8+, p53+, WT1+, ER+, CK5/6+, p63+	TAH-BSO + OT + CTX	Ned at 1 yr

*yr *year, *PAX8 *paired box 8, *WT1 *Wilms tumor 1, *ER *estrogen receptor, *CK5/6 *cytokeratin 5/6, *TAH-BSO *total abdominal hysterectomy and bilateral salpingo-oophorectomy, *OT *omentectomy, *RT *radiation therapy, *CTX *chemotherapy, *na *not available, *ned *no evidence of disease.

In summary, OSC with extensive squamous differentiation is a rare, and the inter- and intratumor heterogeneity may be the reason for this phenomenon. Tumor cells show differences in proliferation, metabolic gene expression, motility, and metastatic potential, and have distinct morphological and phenotypic profiles [13]. Histological diagnostic criteria and biological behavior have not been fully established because only 4 cases (including this case) have been reported at present. We describe this case to facilitate the resolution of diagnostic problems.

## Data Availability

The datasets during and/or analysed during the current study available from the corresponding author on reasonable request.

## Abbreviations

OSC: Ovarian serous carcinomas
CA125: Cancer antigen 125
PAX8: Paired box 8
WT1: Wilms tumor 1
ER: Estrogen receptor
CK5/6: Cytokeratin 5/6
SCC: Squamous cell carcinoma

## Declarations

### Content for publication

The patient provided written informed consent for publication of potentially identifying images and clinical details.
